# Clinical Readiness of Vital Sign–Based Sepsis Prediction

**DOI:** 10.2196/111475

**Published:** 2026-10-27

**Authors:** Li Li, Song Lei, Chansokhon Ngan

**Affiliations:** 1 Department of Critical Care Medicine, Affiliated Hospital of Sichuan Nursing Vocational College, The Third People’s Hospital of Sichuan Province, Chengdu, Sichuan, China; 2 Department of Cardiology, Affiliated Hospital of Sichuan Nursing Vocational College, The Third People’s Hospital of Sichuan Province, Chengdu, Sichuan, China; 3 Yunnan Chinese Language and Culture College, Yunnan Normal University, No. 768, Juxian Street, Chenggong District, Kunming, Yunnan, 650500, China, 86 19181986394

**Keywords:** sepsis prediction, deep learning, vital signs monitoring, temporal modeling, clinical readiness

I read with interest the study by Yang et al [1], which proposed a lightweight transformer-based model for real-time sepsis prediction using six vital signs. The clinical motivation is laudable, but several methodological and interpretational issues warrant clarification.

First, from the original 40,336 PhysioNet records, only 3766 qualified sequences were retained after filtering for vital-sign completeness [1]. For septic records, the authors required <50% missingness within a 16-hour window around sepsis onset, whereas nonseptic sequences were sampled with missingness ≤50%. This selection favors records with greater temporal completeness, yet the model is promoted for low-resource settings where data sparsity may be substantial. The manuscript also does not clearly specify how residual missing values within retained sequences were handled. Because missingness patterns may themselves carry clinical information and influence model estimates [2], reporting the preprocessing strategy, comparing included and excluded patients, and evaluating performance across varying levels of missingness would better demonstrate robustness.

Second, the validation strategy requires greater transparency. The Methods describe a 4:1 train/test split, whereas Table 2 reports averages from 5-fold cross-validation [1]. Although these approaches are compatible, their relationship is not explained. More importantly, the contrastive-learning margins were selected from three candidate configurations according to the lowest mean absolute error (MAE), while Figure 3 tracks area under the receiver operating characteristic curve, area under the precision-recall curve, and MAE on the “test dataset” across training epochs. To avoid potentially optimistic performance estimates, it would be important to clarify whether hyperparameter and epoch-related model-selection decisions were made independently of the data used for final performance estimation. Nested validation or a strictly held-out test set would provide a clearer estimate of generalizability and would address concerns emphasized by contemporary reporting and risk-of-bias frameworks such as TRIPOD+AI (Transparent Reporting of a Multivariable Prediction Model for Individual Prognosis or Diagnosis + AI) and PROBAST+AI (Prediction Model Risk of Bias Assessment Tool + AI) [3,4].

Third, the interpretation of prediction timing requires clarification. The abstract reports a normalized MAE of 0.11%, whereas Table 2 of the original study reports 0.11 and the Methods define MAE as prediction error normalized by input-sequence length [1]. If 0.11 represents a proportion, it corresponds to 11% rather than 0.11% of sequence length. Because input sequences are variable in length, this normalized metric does not directly indicate how many hours before or after sepsis onset predictions occurred. Reporting absolute timing error and clinically interpretable warning time in hours would therefore substantially improve interpretation. The probability threshold used to derive the reported sensitivity (89.29%) and specificity (73%) should also be specified.

These clarifications would not diminish the technical contribution of the Multi-Scale Temporal-aware Contrastive Learning (MSTCL) model but would strengthen its reproducibility, transportability, and clinical readiness.

## Abbreviations

MAE: mean absolute error
MSTCL: Multi-Scale Temporal-aware Contrastive Learning
PROBAST+AI: Prediction Model Risk of Bias Assessment Tool + AI
TRIPOD+AI: Transparent Reporting of a Multivariable Prediction Model for Individual Prognosis or Diagnosis + AI
